# Retinoic acid modulates intrahippocampal levels of corticosterone in middle-aged mice: consequences on hippocampal plasticity and contextual memory

**DOI:** 10.3389/fnagi.2014.00006

**Published:** 2014-02-07

**Authors:** Damien Bonhomme, Véronique Pallet, Gaelle Dominguez, Laure Servant, Nadia Henkous, Pauline Lafenêtre, Paul Higueret, Daniel Béracochéa, Katia Touyarot

**Affiliations:** ^1^INRA, Nutrition et Neurobiologie Intégrée (NutriNeuro), UMR 1286Bordeaux, France; ^2^Université de Bordeaux, Nutrition et Neurobiologie Intégrée (NutriNeuro), UMR 1286Bordeaux, France; ^3^CNRS, Intititut de Neurosciences Cognitives et Intégratives d’Aquitaine, UMR 5287Talence, France; ^4^INSERM, U-930, Université François RabelaisTours, France

**Keywords:** vitamin A, retinoic acid, corticosterone, memory, hippocampal plasticity

## Abstract

It is now established that vitamin A and its derivatives, retinoic acid (RA), are required for cognitive functions in adulthood. RA hyposignaling and hyperactivity of glucocorticoid (GC) pathway appear concomitantly during aging and would contribute to the deterioration of hippocampal synaptic plasticity and functions. Furthermore, recent data have evidenced counteracting effects of retinoids on GC signaling pathway. In the present study, we addressed the following issue: whether the stimulation of RA pathway could modulate intrahippocampal corticosterone (CORT) levels in middle-aged mice and thereby impact on hippocampal plasticity and cognitive functions. We firstly investigated the effects of vitamin A supplementation and RA treatment in middle-aged mice, on contextual serial discrimination task, a paradigm which allows the detection of early signs of age-related hippocampal-dependent memory dysfunction. We then measured intrahippocampal CORT concentrations by microdialysis before and after a novelty-induced stress. Our results show that both RA treatment and vitamin A supplementation improve “episodic-like” memory in middle-aged mice but RA treatment appears to be more efficient. Moreover, we show that the beneficial effect of RA on memory is associated to an increase in hippocampal PSD-95 expression. In addition, intrahippocampal CORT levels are reduced after novelty-induced stress in RA-treated animals. This effect cannot be related to a modulation of hippocampal 11β-HSD1 expression. Interestingly, RA treatment induces a modulation of RA receptors RARα and RARβ expression in middle-aged mice, a finding which has been correlated with the amplitude of intrahippocampal CORT levels after novelty-induced stress. Taken together, our results suggest that the preventive action of RA treatment on age-related memory deficits in middle-aged mice could be, at least in part, due to an inhibitory effect of retinoids on GC activity.

## INTRODUCTION

Vitamin A, through its active metabolite retinoic acid (RA), plays a key role in cognitive functions and more specifically in hippocampus-dependent memory (Lane and Bailey, 2005; McCaffery et al., 2006; Olson and Mello, 2010). Hippocampal function in memory is dependent on plastic changes in synaptic strength, numbers of synapses and neurons, all of which can be modulated by RA (McCaffery et al., 2006). RA regulates gene expression including plasticity-related genes through binding to specific nuclear receptors: RA receptors (RARs) or retinoic X receptors (RXRs; Marill et al., 2003). When the RARs are functionally removed by mutation in mice, deficits both in long-term potentiation and in hippocampal-dependent memory tasks are observed (Chiang et al., 1998; Wietrzych et al., 2005). These deleterious effects have been also evidenced by using the vitamin A deficiency (VAD) model in rats and mice, a nutritional approach leading to a specific hyposignaling of RA signaling pathway (Cocco et al., 2002; Etchamendy et al., 2003; Bonnet et al., 2008). The disruption of retinoid signaling pathway that result from a hypoexpression of some retinoid receptors in target tissues including the hippocampus, occurs naturally during aging in rodents and humans (Enderlin et al., 1997; Etchamendy et al., 2001; Feart et al., 2005; Mingaud et al., 2008; Touyarot et al., 2013). Moreover, it is well established that this RA hyposignaling contributes to the deterioration of hippocampal plasticity and memory processes during aging and these deleterious effects could be reversed by a vitamin A supplementation or RA treatment (Etchamendy et al., 2001; Mingaud et al., 2008; Touyarot et al., 2013).

Although the involvement of retinoids in the modulation of hippocampal plasticity and functions has been well described in aged rodents, molecular mechanisms underlying these effects remain unclear. The hippocampus is a prime target for glucocorticoids (GCs) which bind to abundant mineralocorticoid and GC receptors (MRs and GRs, respectively), known to be involved in the control of hippocampal plasticity and functions (Oitzl and de Kloet, 1992). During aging, hyposignaling of vitamin A pathway occurs concomitantly with hyperactivity of GC pathway leading to excess plasma levels of GCs [cortisol for humans and corticosterone (CORT) for rodents; Born et al., 1995; Tronche et al., 2010] probably due to alterations in the regulation of hypothalamic–pituitary–adrenal (HPA) axis (Mizoguchi et al., 2009). However, although elevated levels of plasma CORT have been largely hypothesized to contribute to age-related memory decline (Cameron and Gould, 1994; Lupien et al., 1998; Yau and Seckl, 2012), few studies focus on the measurement of intrahippocampal CORT levels in aged rodents in relation to memory retrieval performance. Interestingly, it has been recently shown that stress-induced intrahippocampal CORT rise in middle-aged rats was associated with memory impairments in a hippocampal-dependent memory task (Chauveau et al., 2009b) that has been previously shown to be affected in middle-aged mice (Celerier et al., 2004; Beracochea et al., 2008a,b; Chauveau et al., 2009a,b; Pierard et al., 2009; Tronche et al., 2010). The magnitude of intracellular CORT action in the rodent hippocampus is thought to be determined by the hippocampal activity of 11β-hydroxysteroid dehydrogenase type 1 (11β-HSD1), an enzyme that regenerates active CORT within cells, but also by free CORT circulating in blood and delivered to the brain (Seckl, 1997). Thus, both the hyperactivity of hippocampal 11β-HSD1 and the alterations of HPA axis leading to elevated plasma CORT, have been correlated with impairments in hippocampal-dependent memory tasks during aging (Yau et al., 2001; Holmes et al., 2010; Yau and Seckl, 2012).

While the antagonism between GCs and RA pathways has been suggested in few studies using different cell types (Paez-Pereda et al., 2001; Aubry and Odermatt, 2009; Brossaud et al., 2013), no study has scrutinized the regulation of GCs activity by retinoids and its impact on cognitive functions in aged animals. Interestingly, inhibitory effects of RA and vitamin A supplementation have been evidenced on the expression and the activity of 11β-HSD1, in differentiated C2C12 myotubes (Aubry and Odermatt, 2009) and in obese rats liver (Sakamuri et al., 2011). Finally, it has been recently evidenced an increased HPA axis activity in basal and stress conditions associated with an up-regulation of the hippocampal expression of 11β-HSD1 in vitamin A deficient LOU/C rats, which have been normalized by RA treatment (Arvy et al., 2013).

Altogether, these data suggested counteracting effects between RA and GCs pathways. In the present study, we addressed whether the stimulation of RA pathway could modulate intrahippocampal CORT levels in middle-aged mice and thereby modify hippocampal plasticity and cognitive functions. We first compared the effects of vitamin A supplementation (20 IU/g retinol, 2 months) and RA treatment (150 μg/kg, 5 days) on memory retrieval of the contextual serial discrimination (CSD) task in middle-aged mice. Thus, we show that short-term RA treatment in middle-aged mice induced an improvement on memory retrieval associated with an increased hippocampal PSD-95 (postsynaptic scaffolding protein 95 kDa) mRNA expression, used as a marker of synaptic plasticity. Hence, we measured intrahippocampal CORT concentrations (by microdialysis), in basal conditions and after CSD box-induced stress. We demonstrated for the first time that RA treatment significantly decreased and delayed the stress-induced increase of hippocampal CORT levels observed in middle-aged mice without any effect on basal concentration. Therefore, our results strengthened the hypothesis of a close relationship between vitamin A status and GCs-induced cognitive impairments in middle-aged mice. Thus, acting on vitamin A status could be a good strategy to prevent excess GCs-induced cognitive decline occurring with aging.

## MATERIALS AND METHODS

### ANIMALS

Middle-aged (Mid-age) C57BL/6 Jico mice (12-month-old) were purchased from Janvier (Le Genest-Saint-Isle, France). They were housed in a collective cage in a room with a constant airflow system, controlled temperature (21–23°C), and a 12-h light/dark cycle. Mice were granted *ad libitum* access to food and water and were randomly divided into two experimental groups. One group (*n* = 22) was fed with a control diet containing 5 IU retinol/g or a vitamin A-enriched diet containing 20 IU retinol/g during 2 months (INRA, Jouy-en-Josas). The second group (*n* = 39) was fed with a control diet containing 5 IU retinol/g (INRA, Jouy-en-Josas) during 2 months and received subcutaneous injections of RA or vehicle. One week prior to the experiments, all animals were individually housed until sacrifice. Mice were handled and deprived during this period: all subjects were daily weighed in order to maintain 90% of their *ad libitum* body weight throughout the behavioral study. All experiments were performed in accordance with the European Communities Council Directives (86/609/EEC) and the French National Committee (87/848) recommendations, and have been approved by the Animal Care and Use Committee of Bordeaux under the N°50120169-A.

### DIET

A first group of middle-aged mice (Mid-age, *n* = 12) was fed with a control diet containing 5 IU retinol/g (INRA, Jouy-en-Josas) during 2 months. The second half (Mid-age + Vitamin A, *n* = 10) received the vitamin A-enriched diet (20 IU retinol/g) during the same period. The composition of the control diet was the same as the vitamin A-enriched diet, except for retinol content. The 20 IU diet was chosen on the basis of literature as a moderate supplementation to avoid potential toxicity of hypervitaminosis (for review, see Penniston and Tanumihardjo, 2006). Diets started as soon as mice arrived in the laboratory (i.e., at the age of 12–13 months) and continued throughout the entire experiment.

### RA TREATMENT

Middle-aged treated group (12-month-old, *n* = 39) was fed with a control diet containing 5 IU retinol/g (INRA, Jouy-en-Josas) during 2 months and divided into two groups: one group received daily subcutaneous injections of the vehicle (Mid-age + vehicle, *n* = 20), and the second group received RA treatment (Mid-age + RA, *n* = 19). RA (150 μg of *all-trans*-RA/kg body weight, Sigma, France) was dissolved in a mixture (vehicle) containing polyethylene glycol–NaCl–ethanol (70:20:10, by volume) and injected during 5 days. This dose of RA was shown to be effective in reversing age-related hypoexpression of brain signaling and its associated memory impairment in mice (Etchamendy et al., 2001).

### EXPERIMENTAL DESIGN

#### Experimental design displayed in **Figure 1**

In the first experiment, we tested the effects of 2 months of nutritional vitamin A supplementation administered to 12-month-old mice on memory in the CSD task. Thus, after 2 months of diet, middle-aged mice (Mid-age, *n* = 12) and enriched middle-aged mice (Mid-age + Vitamin A, *n* = 10) were tested in the CSD task.

**FIGURE 1:**
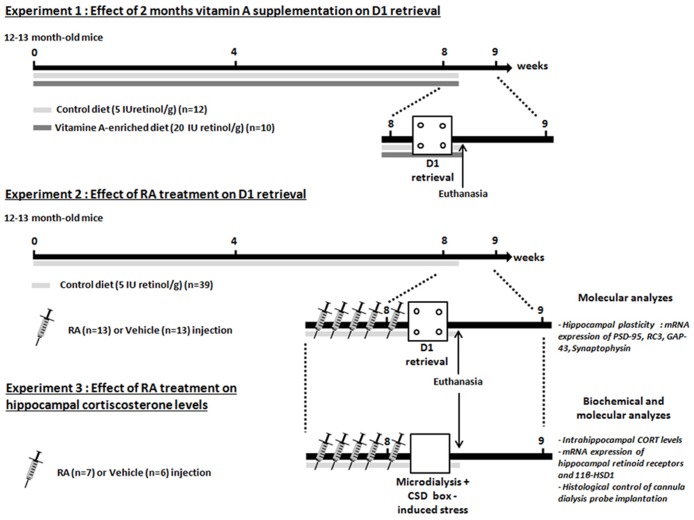
**Experimental design.** Three experiments are carried out. Experiments 1 and 2 are designed to compare the effects of vitamin A supplementation and RA treatment on memory retrieval in the contextual serial discrimination (CSD) task. The Experiment 3 aims to determine the effects of RA treatment on hippocampal CORT relevels in novelty-induced stress. At the end of the Experiments 2 and 3, animals are euthanized, hippocampi and half-brain were dissected in order to carry out RT-PCR measurement (Experiments 2 and 3) and histological control of guide cannula implantation (Experiment 3). The arrows and the bars indicate the time spent and the diet received, respectively. Each needle represented one daily injection of RA or vehicle treatment.

In the second experiment, we tested the effects of RA treatment during 5 days started at middle-age (14-month-old) on memory and hippocampal plasticity. Mice were divided into two groups: mice injected with vehicle are referred to as Mid-age + vehicle (*n* = 13) and mice injected with RA are referred to as Mid-age + RA (*n* = 13). Mice were injected 4 days (one injection/day) before the acquisition phase of the CSD task and until the test phase which occurred 24-h later. Twenty minutes after the end of the test phase, all groups were sacrificed and hippocampi were quickly dissected in order to study the expression of plasticity-related RA target genes by quantitative RT-qPCR [Synaptophysin, RC3, GAP-43 (growth-associated protein 43 or neuromodulin), PSD-95].

In the third experiment, the effects of RA treatment were studied on hippocampal CORT release after CSD box-induced stress in 14-month-old mice. Similarly as in Experiment 2, mice were divided into Mid-age + RA (*n* = 7) and Mid-age + vehicle (*n* = 6). After five consecutive days of injections, at the same time of behavioral testing, microdialysis experiment was performed. After a basal period which lasted 60 min in the microdialysis bowl, animals were placed in the CSD-like box for 10 min and cerebrospinal fluid (CSF) was collected during 105 min post-stress period. Twenty-four hours later, animals were sacrificed and hippocampi were collected in order to evaluate the expression of retinoid receptors (RARs) and GCs-related RA target genes as 11β-HSD1.

#### Contextual serial discrimination task apparatus

The CSD task has been extensively described in earlier studies (Celerier et al., 2004; Beracochea et al., 2008a,b; Chauveau et al., 2009a,b). All tests were performed in a 4-hole board apparatus (45 cm × 45 cm × 30 cm high) enclosed by gray Plexiglas. The 4-hole board apparatus was placed on the floor of the room (3.0 m × 3.0 m × 2.40 m high). The floor of the board was interchangeable (white and rough; black and smooth). On the floor, four holes opening on a food cup (3 cm diameter, 2.5 cm in depth) were located 6 cm away from the side-walls. The apparatus was placed in a room exposed to a 60-dB background noise and a light centered over the apparatus provided 20 lux intensity at the position of the apparatus. The apparatus was cleaned with 95% ethanol, then with water before each mouse behavioral testing. Data were automatically monitored by photoelectric cells and video recording.

#### Acquisition phase

**FIGURE 2:**
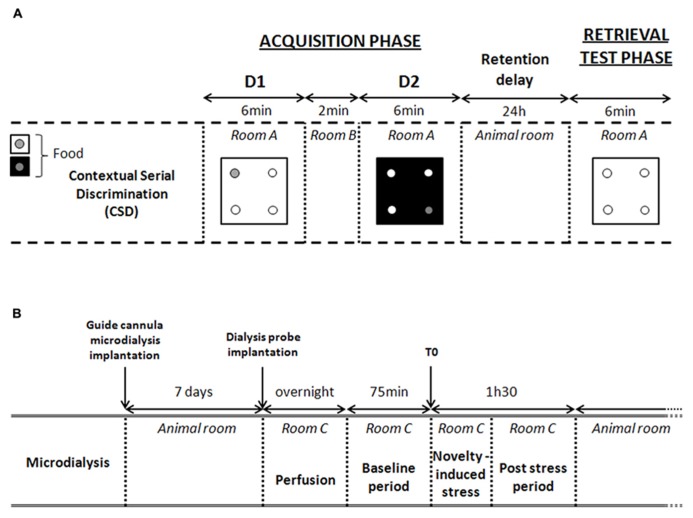
**Experimental procedures.** **(A)** Experimental procedure of the contextual serial discrimination (CSD) behavioral task. D1, discrimination 1; D2, discrimination 2. During retrieval test phase, each mouse is evaluated on D1 floor without food reward. **(B)** Detailed description of microdialysis procedure. Time 0 (T0) corresponds to the last point of the basal period (pre-stress) just before the beginning of the exploration in the CSD-like box. This novelty-induced stress induces an increased hippocampal CORT levels. Thus, microdialysates collected at time 15 (post-stress) corresponds to the CORT levels released between 0 and 15 min after stress.

In the CSD, the acquisition phase took place in room A where animals learned two consecutive spatial discriminations (D1 and D2; see **Figure 2**). Both discriminations differed in terms of the color and texture of the floor (internal context of the 4-hole board) and were separated by a 2-min delay interval. During this delay, the mouse was returned to its home cage in room B. At the beginning of acquisition and retrieval phases, mice were placed in the center of the 4-hole board in an opaque PVC tube for 5 s to provide the animal with a random start in the apparatus. For D1, ten 20-mg food pellets were available only in one of the four holes on the board for 6 min. Location of the baited hole for D1 was randomized for each subject. For D2, ten 20-mg food pellets were consistently located in the opposite symmetrical hole, for 6 min likewise. The environmental spatial cues were made of colored and striped paper sheets positioned at 1.00 m above the 4-hole board. These allocentric cues remained at the same place for both D1 and D2 discriminations and for the retrieval phase. Thus, both discriminations D1 and D2 differed only by way of the internal (floor) contextual cues. Both floors were positioned in a mixed random order during the acquisition of the first and second discrimination tasks. At the end of the acquisition phase, mice returned to their home cage in the animal room for 24 h. Only mice having eaten all the pellets during both acquisition sessions were used for the retrieval test phase.

#### Test phase

Twenty-four hours after the acquisition phase, mice were replaced in the 4-hole board (room A) without any pellets in the apparatus. Mice were placed on the D1 floor and were allowed to freely explore the apparatus for 6 min. Performance was assessed by measuring the number of head-dips in each hole during 6 min.

#### Measurements

Memory retrieval performance was evaluated through the exploration rates into the different holes. Correct responses were defined as head-dips into the hole previously baited on the same floor-context during the acquisition phase, and were calculated as follows: (head-dips into the baited hole/total number of head-dips in the four holes) × 100. Interfering responses were defined as head-dips into the hole previously baited on the other floor-context in the acquisition phase and were calculated on same manner as correct responses. Chance level was 25%.

### SURGERY AND HISTOLOGY

For Experiment 3, 1 week before the microdialysis study, mice were anesthetized with a ketamine (100 mg/kg body weight)–xylazine (10 mg/kg body weight) solution and placed into a stereotaxic frame. A single guide cannula microdialysis (CMA/7 Microdialysis probe, CMA Microdialysis, Sweden) was implanted randomly in the left or the right hemisphere, in the bottom of the parietal cortex at the following coordinates from the bregma (Paxinos and Franklin, 2001): anteroposterior = -2000 μm, lateral = ±1400 μm, and vertical = -900 μm. The guide cannula was fixed with dental cement to the skull. All operated mice were allowed to recover in their home cages in the animal room for at least 7 days before the microdialysis experiment. On the day of experiment, the microdialysis probe was lowered 1 mm below through the guide cannula so that the microdialysis membrane is located into the dorsal hippocampus. At the end of the microdialysis experiment, mice were euthanatized and the half of the brain containing the guide cannula was dissected, washed into a saline solution (NaCl 0.9%) and postfixed in a 4% paraformaldehyde solution. After 3 weeks postfixation period, 50 μm frontal sections were cut on a vibratome (Leica). A cresyl violet stain was used to locate the microdialysis probe with utmost accuracy.

### *IN VIVO* MICRODIALYSIS

Seven days after surgery, a dialysis probe (CMA/7; CMA Microdialysis AB, Sweden; length: 1 mm; molecular cut-off 6 kDa and membrane diameter: 0.24 mm outer diameter) was carefully implanted into the right or left dorsal hippocampus. Mice were then individually housed in a system allowing animals to move freely (CMA/120; CMA Microdialysis AB, Sweden) overnight. After the overnight perfusion at 0.5 μL/min, freely moving animals were continuously perfused with a sterile-filtered saline solution (Dulbecco’s phosphate buffered saline; SIGMA; in g/L: CaCl_2_, 0.133; MgCl_2_, 0.1; KCl, 0.2; KH_2_PO_4_, 0.2; NaCl 8.0; Na_2_HPO_4_, 1.15; pH between 7.1 and 7.5) at a 1-μL/min flow rate through a micro-infusion pump. Baseline dialysates were collected for 60 min before novelty-induced stress task. During the novelty-induced stress, animals were carefully placed in an open field square box similar to the CSD box (45 cm × 45 cm × 30 cm high). After 10 min exploration, animals were then relocated in the microdialysis freely moving chamber for 95 min. Microdialysates were sampled every 15 min using tubes with a dead volume of 1.2 μL/100 mm length (CMA Microdialysis AB). Samples were stored at 80°C. Free CORT levels measured in the dialysates (in nM) were expressed as the percentage of the averaged baseline values collected before the injection.

### INTRA-HIPPOCAMPAL CORT ASSAY

A commercially prepared Enzyme Immunoassay kit was used to measure HPC CORT concentrations in the microdialysates (Correlate-EIA^TM^, Assay Designs, Ann Arbor, USA) according to the manufacturer’s instructions. The sensitivity of the assay was 0.08 nmol/L. Therefore, baseline sample concentration was more than 10-fold superior than sensitivity threshold.

### REAL-TIME qPCR ANALYSIS OF TARGET GENE EXPRESSION IN THE HIPPOCAMPUS

The other half of the hippocampus was used to measure gene expression. Extraction of RNA was conducted using an extraction kit (TRIzol reagent, Invitrogen, Saint Aubin, France) according to the manufacturer’s instructions. The integrity of the purified RNA was verified using the RNA 6000 Nano LabChip kit in combination with the 2100 bioanalyzer (Agilent Technologies). The concentrations of RNA were determined by using a nanodrop (ND-1000; Labtech). Using OligodT and random primers (Promega, Charbonnières-les-Bains, France), cDNA was synthesized from 1 μg of RNA with ImPromII reverse transcriptase (Promega, Charbonnières-les-Bains, France) according to the manufacturer protocol. The real-time qPCR was performed using the LightCycler 480 system with a 96-well format (Roche Diagnostics) in a volume, containing 1× LightCycler 480 SYBR Green I Master solution, 0.5 μM of each primer and 6 μL of cDNA. The forward and reverse primer sequences are summarized in **Table 1**. The results are expressed as the target/reference ratio divided by the target/reference ratio of the calibrator.

**Table 1:** Primers used for LightCycler RT-qPCR.

Gene name	Nucleotide sequence 5′–3′	Product length (bp)
GAPDH	F: CCAGTGAGCTTCCCGTTCA	78
	R: GAACATCATCCCTGCATCCA	
Actin	F: AAAACGCAGCTCAGTAACAGTCC	220
	R: AGGATGCAGAAGGAGATTACTGC	
Synaptophysin	F: TCCTTGCATGTGTTTCCTGTCTG	137
	R: GCAGTGTTCGCTTTCATGTGG	
PSD-95	F: TGGATCACAGGGTCGAGAAG	101
	R: CTTTGGTAGGCCCAAGGATG	
RC3	F: CACTCTCCGCTCTTTATCTTCTTC	127
	R: GCTCCAAGCCAGACGACGATATTC	
GAP-43	F: GGGGAGTTATCAGTGGTAGC	255
	R: GTGATGCACCAGCTGCTGAGG	
RARα	F: GGCGAACTCCACAGTCTTAATG	118
	R: GCTGGGCAAGTACACTACGAAC	
RARβ	F: GGGGTATACCTGGTACAAATTCTGA	227
	R: CAGCTGGGTAAATACACCACGAA	
RARγ	F: CCCAAGGATGCTGATGAAAATC	63
	R: GCCCTTTCTGCTCCCTTAGTG	
11β-HSD1	F: GGAAGGTCTCCAGAAGGTAGTGTC	51
	R: GAGGCTGCTCCGAGTTCAAG	

*Sequences are shown for forward (F) and reverse (R) primers. GAPDH, glyceraldehyde-3-phosphate dehydrogenase-like; PSD-95, postsynaptic density protein 95; RC3, neurogranin; GAP-43, growth associated protein 43; RARs, retinoic acid receptors; 11β-HSD1, 11-beta-hydroxysteroid dehydrogenase type 1.*

### STATISTICAL ANALYSIS

Contextual serial discrimination test and RT-qPCR data were analyzed by a one-way analysis of variance (ANOVA): effect of vitamin A or RA treatment. Comparisons of retrieval performance with chance level were calculated with a one sample *t*-test (with hypothesized mean = chance level = 25%). Hippocampal CORT release was analyzed using a two-way ANOVA with repeated measures: effect of RA treatment and Time. All results were expressed as mean ± SEM.

## RESULTS

### EFFECTS OF VITAMIN A SUPPLEMENTATION AND SHORT-TERM RA TREATMENT ON CONTEXTUAL SERIAL DISCRIMINATION MEMORY

**FIGURE 3:**
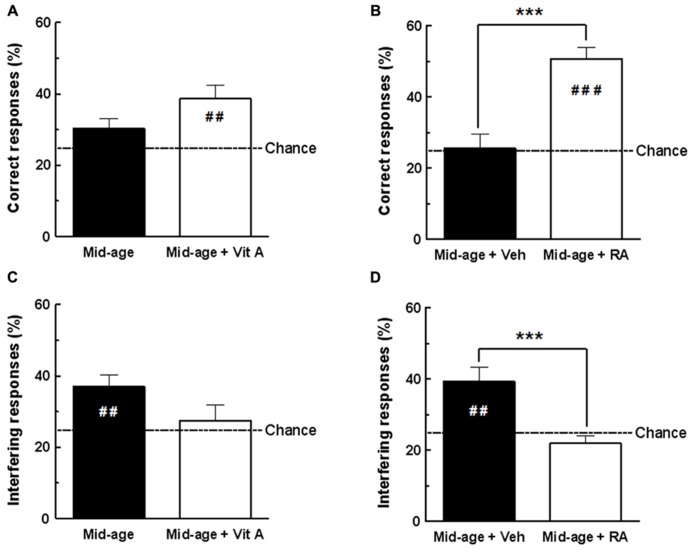
**Effects of vitamin A supplementation and RA treatment on D1 retrieval.** **(A)** The percentage of correct responses for vitamin A supplemented group was different from the chance level (25%) compared to control group. **(B)** The percentage of correct responses for RA-treated group was increased compared to vehicle and different from chance level. **(C)** The percentage of interfering responses for vitamin A supplemented group was not significantly different from chance level compared to control group. **(D)** Percentage of interfering responses for RA-treated group was significantly decreased compared to vehicle and was not different from chance level. ***p < 0.001 vs Control by one-way ANOVA; ^##^*p* < 0.01 vs Chance, ^###^*p* < 0.001 vs Chance, by one sample *t*-test. *n* = 10–13 per group.

In the present experiment, we compared the effects of 2 months of vitamin A supplementation and short-term RA treatment on D1 retrieval in middle-aged mice (**Figure 3**).

#### Acquisition phase

The acquisition phase of groups tested for D1 was analyzed according to the further random attribution of mice to different diet or treatment conditions (Control vs Vitamin A, Vehicle vs RA). No between-group difference was observed on behavioral measures (number of head-dips into baited holes, total number of head-dips; n.s. in all comparisons – data not shown).

#### Retrieval test phase – correct responses

As shown in **Figure 3A**, one-way ANOVA of percentage of correct responses in D1 retrieval test revealed no effect of vitamin A supplementation [*F*(1,20) = 3.23, *p* = 0.087; Mid-age + Vitamin A: 38.59 ± 3.81% vs Mid-age: 30.26 ± 2.80%]. However, the enriched middle-aged group exhibited performances significantly above the chance level (different from 25%; one sample test *p* < 0.01) compared to control middle-aged mice which performed at chance (one sample test *p* = 0.087, n.s.). As shown in **Figure 3B**, a significant improvement of memory retrieval was observed in middle-aged mice receiving RA treatment compared to the vehicle-treated group [*F*(1,24) = 25.32, *p* < 0.001; Mid-age + RA 50.66 ± 3.16% vs Mid-age + vehicle: 25.68 ± 3.82%]. In addition, D1 performance of RA-treated mice was significantly above the chance level (one sample test *p* < 0.0001) showing that they accurately remember the previously baited hole, in contrast to vehicle-treated mice.

#### Retrieval test phase – interfering responses

During retrieval of D1, interfering responses consisted of exploration into the hole baited on discrimination D2. As shown in **Figure 3C**, one-way ANOVA analyzes revealed that vitamin A supplementation does not affect the percentage of responses in the interfering hole [*F*(1,20) = 3.18, *p* = 0.90; Mid-age + Vitamin A: 27.48 ± 4.29% vs Mid-age 36.98 ± 3.31%]. However, as seen in **Figure 3D**, one-way ANOVA showed that RA-treated mice exhibited significantly less interfering responses than vehicle control mice [*F*(1,24) = 15.18, *p* < 0.001; Mid-age + RA: 21.88 ± 2.20% vs Mid-age + vehicle: 39.42 ± 3.93%]. In addition, the percentage of responses in the interfering hole was above the chance level for the vehicle-treated mice (one sample test *p* = 0.003) confirming impaired contextual retrieval for D1, whereas RA-treated mice performed at chance (one sample test *p* = 0.182, n.s.).

In conclusion, our results demonstrate that both vitamin A supplementation and RA treatment improve memory performances in the CSD task. However, a short-term RA treatment in middle-aged subjects was more efficient to improve retrieval of D1 context than 2 months of vitamin A supplementation. Thus, short-term RA treatment was chosen in our subsequent experiments to study the effects of retinoid pathways stimulation in middle-aged mice on hippocampal CORT levels and hippocampal expression of retinoid-target genes.

### EFFECTS OF RA TREATMENT ON HIPPOCAMPAL EXPRESSION OF PLASTICITY-RELATED GENES

**FIGURE 4:**
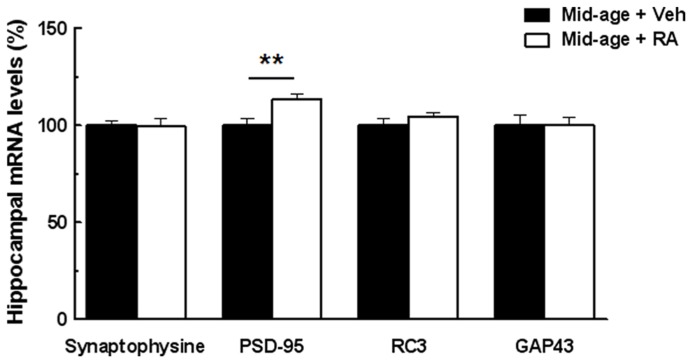
**Effects of RA treatment on mRNA expression of hippocampal plasticity-related genes.** RA treatment significantly increases PSD-95 mRNA levels. No difference between groups was detected for Synaptophysin, RC3, and GAP-43 mRNA levels. **p < 0.01 vs Control; by one-way ANOVA, *n* = 6 per group.

In order to study the possible neurobiological mechanisms underlying the improvement of memory performances in RA-treated mice, we tested whether RA treatment can modulate expression of some hippocampal plasticity genes such as synaptophysin, PSD-95, RC3 (neurogranin), and GAP-43, measured 20 min after the end of the CSD task. As shown in **Figure 4**, one-way ANOVA performed on mRNA expression of hippocampal PSD-95 revealed an effect of RA treatment [*F*(1,9) = 10.46, *p* = 0.01]. Indeed, an increase of PSD-95 expression (+13%) was found in RA-treated mice. However, no effect of RA treatment was found on the mRNA expression of synaptophysin, RC3, and GAP-43 [*F*(1,9) = 0.008, n.s., *F*(1,9) = 1.90, n.s., and *F*(1,9) = 0.009, n.s., respectively].

### EFFECTS OF RA TREATMENT ON HIPPOCAMPAL CORT RELEASE

We studied the involvement of hippocampal CORT in the effects of RA treatment on hippocampal plasticity and functions in middle-aged mice. Thus, CORT levels in hippocampal dialyzates were analyzed in basal conditions and after CSD box-induced stress according to the further random attribution of mice to vehicle or RA-treated groups.

**FIGURE 5:**
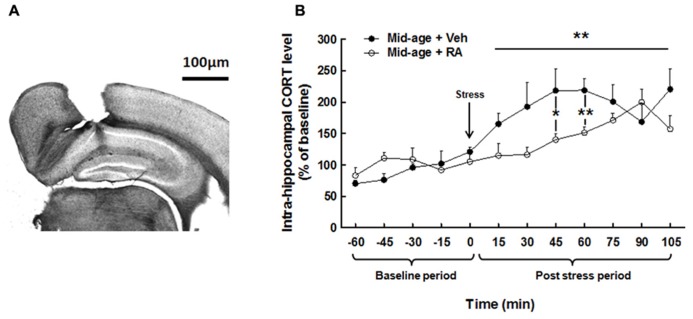
**Effects of RA treatment on hippocampal CORT release.** **(A)** Histological control of intra-hippocampal microdialysis probe implantation. Photomicrograph (×20) of coronal brain section stained with cresyl violet. Stereotaxic coordinates from bregma: AP = -2000 μm, L = ±1400 μm, and V = -900 μm. **(B)** Hippocampal CORT concentration was similar for all groups during basal period. During post-stress period, RA-treated group showed a decrease in hippocampal CORT levels particularly at 45 and 60 min after stress. *p < 0.05, **p < 0.01, by two-way ANOVA with repeated measures and one-way ANOVA, *n* = 6–7 per group.

As shown in **Figure 5A**, a photomicrograph of coronal brain section stained with cresyl violet from a typical mouse shows that microdialysis probe were located inside the dorsal hippocampus, between CA1 and DG areas. **Figure 5B** represents CORT levels in the dorsal hippocampus and results are expressed in percentage of variation of baseline.

#### Basal hippocampal corticosterone levels

Two-way ANOVA on hippocampal CORT levels (**Figure 5B**) along the basal period (-60 to 0 min) evidenced no global effect of treatment [*F*(1,11) = 1.77, n.s.], nor interaction treatment × time [*F*(4,44) = 1.00, n.s.] showing no differences in basal hippocampal CORT between groups (Mid-age + vehicle: 1.11 ± 0.20 nM vs Mid-age + RA: 1.61 ± 0.37 nM.)

#### Hippocampal corticosterone levels after CSD box-induced stress

Two-way ANOVA performed on hippocampal CORT released along the post-stress period (from 0 to 105 min) revealed a global effect of RA treatment [*F*(1,11) = 4.54, *p* = 0.05], an effect of time [*F*(7,77) = 4.43, *p* < 0.0001] with an interaction treatment × time [*F*(7,77) = 2.47, *p* < 0.05]. Firstly, as compared to the last pre-stress sample (121.08 ± 7.66%; time 0), stress induced a fast and rapid increase in CORT levels from 15 min after stress (165.36 ± 16.58%) to 105 min (220.55 ± 32.15%) in vehicle-treated mice. Moreover, a faster increase in CORT levels was observed 15 min after CSD box-induced stress in control middle-aged mice as compared to RA-treated middle-aged mice [*F*(1,11) = 3.91, *p* = 0.07; Mid-age + vehicle: 165.36 ± 16.58% vs Mid-age + RA: 114.93 ± 18.80%] as well as 30 min [*F*(1,11) = 4.04, *p* = 0.07; Mid-age + vehicle: 192.71 ± 38.7% vs Mid-age + RA: 116.50 ± 11.97%], 45 min [*F*(1,11) = 5.56, *p* < 0.05; -78% between Mid-age + vehicle: 218.48 ± 34.42% and Mid-age + RA: 140.26 ± 9.22%] and 60 min [*F*(1,11) = 11.43, *p* < 0.01; -77% between Mid-age + vehicle: 218.8 ± 18.88% and Mid-age + RA: 151.36% ± 9.05%] after stress delivery.

Thus, CSD box-induced stress significantly increased hippocampal CORT levels in middle-aged rats but the stress-induced CORT rise was lower and delayed for an extended period of time in RA-treated middle-aged mice.

### EFFECTS OF RA TREATMENT ON HIPPOCAMPAL EXPRESSION OF RETINOID RECEPTORS AND 11β-HSD1

In order to study the relationships between retinoid and GC pathway, hippocampal expression of RARs and 11β-HSD1 was assessed in middle-aged mice. For gene expression, results are expressed in percentage of variation by comparison to vehicle-treated group.

**FIGURE 6:**
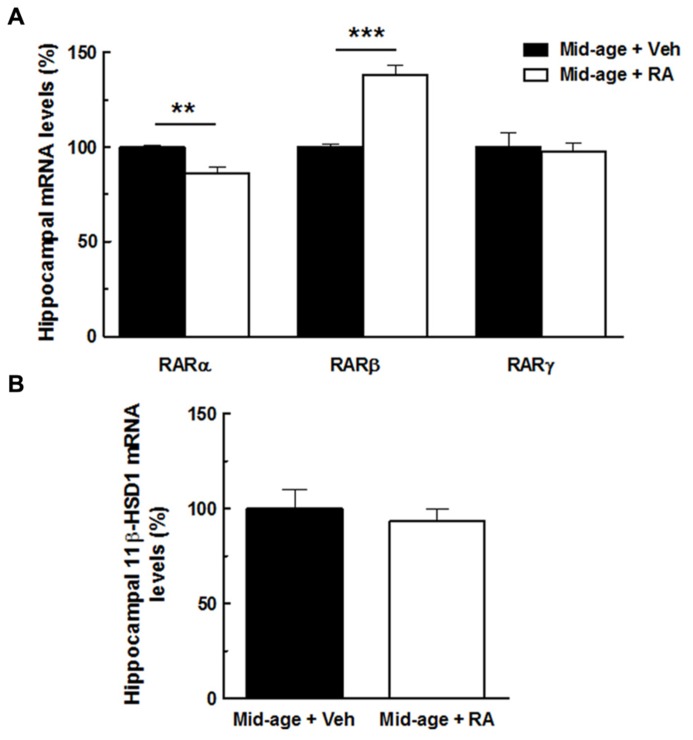
**Effects of RA treatment on hippocampal expression of RARs (RARα, RARβ, RARγ) and 11β-HSD1.** **(A)** The percentage of RARα mRNA expression was decreased by RA treatment whereas the percentage of RARβ mRNA expression was increased by the treatment. No effect on RARγ expression was found. **(B)** RA treatment did not modulate hippocampal mRNA levels of 11β-HSD1. By one-way ANOVA, *n* = 6–7 per group. ***p < 0.001 vs Mid-age + Veh; **p < 0.01 vs Mid-age + Veh, by one-way ANOVA, *n* = 6–7 per group.

As seen in **Figure 6A**, one-way ANOVA performed on hippocampal RARα mRNA expression showed an effect of RA treatment [*F*(1,10) = 19.16, *p* < 0.01]. Indeed, RA treatment induced a decreased RARα expression (-14%) compared to vehicle-treated mice. By contrast, hippocampal RARβ mRNA expression is increased by 38% compared to vehicle-treated mice [ANOVA, effect of RA treatment, *F*(1,10) = 53.88, *p* < 0.001]. Finally, no effect of RA treatment was detected on hippocampal RARγ expression [*F*(1,10) = 0.55, n.s.]. As shown in **Figure 6B**, hippocampal mRNA expression of 11β-HSD1 was not affected by RA treatment [*F*(1,10) = 0.32, n.s.].

**FIGURE 7:**
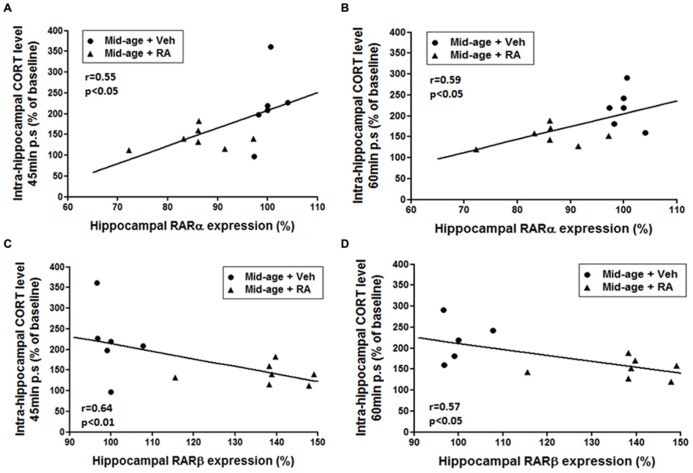
**Relationships between hippocampal expression of RARs (RARα, RARβ) and intrahippocampal CORT levels.** **(A,B)** Correlation analyzes between RARα and CORT levels. Intrahippocampal CORT levels [45 min **(A)** and 60 min **(B)** post-stress] positively correlated with RARα hippocampal mRNA expression (*r* = 0.55, *p* < 0.05 and *r* = 0.59, *p* < 0.05, respectively). **(C,D)** Correlation analyzes between RARβ and CORT levels. Intrahippocampal CORT levels [45 min **(C)** and 60 min **(D)** post-stress] negatively correlated with RARβ hippocampal mRNA expression (*r* = 0.64, *p* < 0.01 and *r* = 0.57, *p* < 0.05, respectively).

Moreover, correlations analyzes were performed between hippocampal expression of RARs and intrahippocampal CORT levels in order to study interactions between RA and GCs signaling pathways (**Figure 7**). Correlation analyzes between hippocampal expression of RARα and CORT levels showed that a decreased expression of RARα was associated to a lower hippocampal CORT release 45 and 60 min post-stress (**Figures 7A,B**, *r* = 0.55, *p* < 0.05 and *r* = 0.59, *p* < 0.05, respectively). Moreover, correlation analyzes revealed a strong negative correlation between intrahippocampal CORT levels and hippocampal expression of RARβ 45 and 60 min post-stress (**Figures 7C,D**, *r* = 0.64, *p* = 0.01 and *r* = 0.57, *p* < 0.05, respectively): low intrahippocampal CORT was associated with elevated expression of RARβ. Finally, no significant correlation was found between hippocampal CORT levels and hippocampal RARγ expression.

Thus, these data sustained the idea that the modulation of RA signaling pathway by RA treatment during mid-life can modulate GCs activity in the hippocampus.

## DISCUSSION

The aim of this study was to explore the involvement of retinoids in the modulation of intrahippocampal CORT levels in middle-aged mice and its impact on hippocampal plasticity and functions. Middle-aged mice treated with five daily consecutive injections of RA retrieve better D1 discrimination in CSD task than mice fed with 2 months of vitamin A supplementation showing that RA treatment seems to be more efficient than vitamin A supplementation. Moreover, we show that the improvement of D1 retrieval by RA treatment is associated with an increased hippocampal PSD-95 mRNA expression, one of plasticity-related RA target genes. Interestingly, RA treatment can decrease and delay the stress-induced increase in dorsal hippocampus CORT rise in middle-aged mice. This effect cannot be related to a modulation of hippocampal 11β-HSD1 expression. In addition, RA treatment induces a modulation of RA receptors RARα and RARβ expression in middle-aged mice, which has been correlated with intrahippocampal CORT levels after CSD-box-induced stress, showing that retinoid signaling could be an important modulator of intrahippocampal GCs activity and thereby hippocampal plasticity and functions.

### EFFECTS OF RETINOIC ACID TREATMENT ON CSD TASK AND HIPPOCAMPAL EXPRESSION OF PLASTICITY-RELATED TARGET GENES AT MIDDLE-AGE

We first explored the effects of vitamin A supplementation and RA treatment on CSD task that allows the detection of early signs of age-related hippocampal-dependent memory deficits (Chauveau et al., 2010; Tronche et al., 2010). Studies in middle-aged mice have important implications for the treatment of the non-pathological age-associated cognitive decline, as well as mild cognitive impairment (Pepeu, 2004). The effects of stimulation of retinoid pathway on memory performance have not yet been tested in middle-aged rodents. Thus, our study demonstrates for the first time that a mid-life RA treatment is efficient to correct “early” age-related hippocampal-dependent memory deficits. The ability of cells to convert vitamin A into RA, the active metabolite, and therefore to initiate retinoid signaling would be altered during aging (Azais-Braesco et al., 1995). Thus, injections of RA at middle-age, would be more efficient than 2 months of nutritional vitamin A supplementation involving metabolization steps. Our present results show that 2 months of vitamin A supplementation with a dose of 20 IU retinol/g is not sufficient to observe preventive effects on memory processes. However, a recent study of our team has shown that the stimulation of retinoid pathway by 4 months of vitamin A supplementation allowed the correction of hippocampal-dependent memory deficits in aged rodents but in this study, the animals were fed with higher content of vitamin A (45 IU retinol/g) and for a longer time which would be an efficient way of improving metabolism (Etchamendy et al., 2001; Drapeau et al., 2003, 2007; Mingaud et al., 2008; Touyarot et al., 2013).

In our current study, the memory retrieval improvement of D1 observed in middle-aged mice after RA treatment can be ascribed to an improvement of hippocampal-dependent memory function. Indeed, mice with lesion of the dorsal hippocampus exhibited an impaired memory retrieval pattern in the CSD task similar to that of middle-aged mice of the present study (Chauveau et al., 2009b). Deficits in hippocampal-dependent tasks reported in middle-aged rats (Frick et al., 1995; Markowska and Breckler, 1999; Kadish et al., 2009), could result from dysfunction of hippocampal plasticity (Kaczorowski and Disterhoft, 2009). Thus, the modulation of hippocampal plasticity, one plausible mechanism underlying hippocampal memory function would be critical for retention of D1 discrimination in the CSD task. The genes we chose to study are hippocampal plasticity-related RA target genes and all major components of presynaptic and postsynaptic density. Thus, we show that RA treatment is efficient not only to improve hippocampal-dependent memory processes but also hippocampal synaptic plasticity as evaluated by the expression of some plasticity-related RA target genes in middle-aged mice. Interestingly, we demonstrate that RA treatment at mid-life increased hippocampal PSD-95 mRNA expression, known to be involved in memory processes (Migaud et al., 1998; Carlisle et al., 2008). PSD-95 recruits and clusters *N*-methyl-D-aspartate (NMDA) and α-amino-3-hydroxy-5-methyl-4-isoxazolepropionic acid (AMPA) receptors, ion channels, cytoskeletal components, and signal transduction molecules in response to synaptic activity (Beique et al., 2006; Vickers et al., 2006). During spatial learning and memory formation, PSD-95 is recruited to synaptic lipid rafts, suggesting that regulated localization as well as expression of this protein is a necessary component of synaptic plasticity (Delint-Ramirez et al., 2008). Alterations in the content of PSD-95 in the hippocampal formation have been observed in aged-learning impaired rats (Nyffeler et al., 2007). In addition, a positive correlation between the decreased expression levels of hippocampal PSD-95 and the decline of performance in a hippocampal-dependent spatial memory task have been shown (VanGuilder et al., 2011). Thus, we proposed that the promotion of hippocampal PSD-95 expression in middle-aged mice treated with RA could be one of the plausible mechanisms by which RA treatment might exert promnesic effects in the CSD task. In addition, we show that RA treatment on middle-aged mice does not modify Synaptophysin, RC3, and GAP-43 hippocampal mRNA expression. It has been demonstrated to date that RA treatment or vitamin A supplementation can increase levels expression of RC3 (Etchamendy et al., 2001) and GAP-43 (Mingaud et al., 2008) during aging. However, no effect of vitamin A supplementation nor RA treatment has been evidenced on hippocampal GAP-43 mRNA expression in middle-aged mice (Mingaud et al., 2008) suggesting thereby that the modulation of retinoid status at mid-life would not be efficient to induce changes in these plasticity-related genes and would be more specific to PSD-95.

### EFFECTS OF RETINOIC ACID TREATMENT ON INTRAHIPPOCAMPAL CORTICOSTERONE LEVELS: INVOLVEMENT OF RETINOIC ACID RECEPTORS

In order to better understand some potential mechanisms involved in the regulation of hippocampal plasticity and memory processes in the CSD task by RA treatment, we have tested whether RA signaling pathway could be a regulator of intrahippocampal CORT levels in middle-aged mice. Our study shows that RA treatment does not influence basal levels of intrahippocampal CORT but reduces and delays the CORT rise after CSD-induced stress in middle-aged mice.

Therefore, in order to establish a link between vitamin A status in middle-aged mice and hippocampal CORT rise after stress conditions, we measured the expression of hippocampal retinoid receptors. We show that the low CORT release in middle-aged RA-treated mice has been associated with a decrease in hippocampal RARα and an increase in RARβ mRNA expression levels thus showing that retinoid signaling could be an important modulator of intrahippocampal GCs activity. It has been shown that the hippocampal expression of retinoic receptors is a good indicator of vitamin A status and intracellular availability of RA. Indeed, several studies have demonstrated age-related decreases in mRNA expression or protein levels of retinoid receptors in mice and rat brain (Enderlin et al., 1997; Etchamendy et al., 2001; Feart et al., 2005; Mingaud et al., 2008; Dyall et al., 2010) indicating a decreased bioavailability of the ligand. More specifically, age-induced decreased RARβ in the whole brain has been associated with memory deficits and all these effects can be normalized by RA treatment (Etchamendy et al., 2001). According to these results, we evidence that RA treatment increases hippocampal expression of RARβ in middle-aged while RARα is decreased. More importantly, we have shown correlations between hippocampal expression of these retinoid receptors and hippocampal CORT levels. Thus, our results suggested that RA treatment-induced up-regulation of hippocampal RARβ but also down-regulation of hippocampal RARα could contribute to limit hippocampal CORT rise. These results further substantiated our hypothesis of a close relationship between hippocampal retinoid and GC pathways.

### POSSIBLE MECHANISMS INVOLVED IN THE EFFECTS OF RA TREATMENT ON INTRAHIPPOCAMPAL CORT LEVELS

#### 11β-HSD1 hypothesis

The magnitude of intracellular GCs action is thought to be determined by the activity of 11β-HSD1 (Seckl, 1997), that is increased during aging in hippocampus and correlated with impaired cognitive performance in the water maze (Holmes et al., 2010). Indeed, 11β-HSD1 regenerates active GCs from their inactive forms, in specific areas in the adult brain, such as the hippocampus, thereby effectively amplifying intracellular GC levels before they bind to MRs and/or GRs (Holmes et al., 2003). Interestingly, inhibitory effects of RA and vitamin A supplementation have been shown on the expression and the activity of 11β-HSD1, in differentiated C2C12 myotubes (Aubry and Odermatt, 2009), in obese rats liver (Sakamuri et al., 2011) and in vitamin A deficient rats hippocampus (Arvy et al., 2013). Thus, we suggest that the effect of RA treatment on hippocampal CORT levels could directly be mediated, through retinoid receptors which act as transcription factors. However, our results show that hippocampal 11β-HSD1 expression is not modulated by RA treatment in middle-aged mice suggesting that other mechanisms would lead to a modulation of hippocampal CORT levels.

#### Modulation of HPA axis

Another hypothesis would be a dysfunctioning of the HPA axis in response to stress in middle-aged mice and a subsequent deregulation of hippocampal CORT levels (Herman et al., 2001). Brain aging has been construed as being conditioned by an excessive plasma GC secretion leading to damages on brain areas, such as hypothalamus or hippocampus, involved in the control of the activity of the HPA axis (Cameron and Gould, 1994; Lupien et al., 1998; Yau and Seckl, 2012). More recently, an increase in HPA axis activity was shown in vitamin A deficient rats LOU/C, exhibiting retinoid hyposignaling pathway, leading to elevated total plasma CORT level in basal and stress conditions, which is restored to control level by RA treatment (Arvy et al., 2013). However, contrary to this result, it has been shown that chronic RA treatment with high doses (2 mg/kg) induced a hyperactivation of HPA axis along with an increased basal plasma CORT levels in young rats (Cai et al., 2010). This discrepancy may be related to differences in treatment used (duration of RA injections, doses) and nutritional status (vitamin A deficient rats vs control rats). Moreover, an interesting recent study, has proposed that excessive RA treatment could induce hyperactivation of HPA axis leading to hypersecretion of plasma CORT through an increased hypothalamic expression of RARα and a deficient negative feedback at the level of hypothalamic GR expression (Hu et al., 2013). In our present study, elevated expression of RARα would also be deleterious and associated with an alteration in HPA axis. We can propose that our more physiologic short-term RA treatment (150 μg/kg) would lead to a normalization of HPA axis activity in middle-aged mice probably via a regulation of hippocampal RARs and GRs levels. However, the precise molecular mechanism by which RA and GCs signaling interacts, awaits future study. Thus, altogether these data suggested that vitamin A status would be an important nutritional factor to take into account in the modulation of the CORT at systemic levels which probably would impact on hippocampal availability of CORT (Yau and Seckl, 2012).

### DOES RETINOIC ACID TREATMENT ACT ON HIPPOCAMPAL PLASTICITY AND FUNCTIONS VIA GLUCOCORTICOIDS?

In the present study, we show that RA treatment has beneficial effects on hippocampal plasticity and functions at middle-age that probably via the modulation of intrahippocampal CORT levels. This hypothesis is also supported by the results of Tronche et al.’s (2010) study showing that the stress-induced hippocampal CORT levels in middle-aged mice were of greater magnitude as compared to young mice, and that a metyrapone (an inhibitor of CORT synthesis) injection before stress blocked both the stress-induced hippocampal CORT rise and associated memory retrieval impairment. GCs can promote behavioral adaptation, regulating (either enhancing or impairing) several memory processes (Joels, 2008). For example, stress can enhance the consolidation of spatial and emotional memories, but it can also impair their retrieval (de Quervain et al., 1998; Sandi and Pinelo-Nava, 2007). Thus, insofar as it has been shown that the effectiveness of learning throughout a continuum of stress and/or CORT levels generally follows an inverted U-shaped function (Salehi et al., 2010), we may hypothesize therefore that RA treatment could normalize stress levels in middle-aged mice during CSD task. Interestingly this hypothesis fits well with a previous study from our group having shown that the administration of diazepam inhibited the stress-induced increase of hippocampal CORT levels and in parallel, restored memory performance in the CSD task (Beracochea et al., 2011). Finally, a recent study has found a decrease in hippocampal PSD-95 protein levels after chronic CORT exposure (Cohen et al., 2011). Thus, PSD-95 seems to be a good plasticity-related RA and GCs target genes that could be involved, at least in part, in the positive effects of RA treatment on hippocampal functions.

## CONCLUSION

The present study provides breakthrough evidence that RA treatment can decrease and delay the stress-induced intrahippocampal CORT rise in middle-aged mice. Moreover, correlations between vitamin A status evaluated by the hippocampal expression of retinoid receptors and CORT levels in the hippocampus have been evidenced. Interestingly, our data suggest that the improvement of memory and hippocampal plasticity in RA-treated mice could result from a decreased hippocampal CORT rise but future investigations are needed to establish a clear-cut causal link. Since vitamin A metabolism alterations occur during aging, our results are of prime importance and further substantiate the view that maintaining a normal vitamin A status in seniors would be crucial to prevent age-related cognitive impairments.

## Conflict of Interest Statement

The authors declare that the research was conducted in the absence of any commercial or financial relationships that could be construed as a potential conflict of interest.
